# Comprehensive analysis of the fecal microbiota of healthy Japanese adults reveals a new bacterial lineage associated with a phenotype characterized by a high frequency of bowel movements and a lean body type

**DOI:** 10.1186/s12866-016-0898-x

**Published:** 2016-11-28

**Authors:** Kaihei Oki, Mutsumi Toyama, Taihei Banno, Osamu Chonan, Yoshimi Benno, Koichi Watanabe

**Affiliations:** 1 Yakult Central Institute, 5-11 Izumi, Kunitachi, Tokyo 186-8650 Japan; 2 Benno Laboratory, RIKEN Innovation Center, 2-1, Hirosawa, Wako, Saitama, 351-0198 Japan

**Keywords:** Human gut microbiota, Healthy Japanese adult, Bowel movement frequency, Body mass index (BMI), 16S metagenomics, Dietary habits and daily routine

## Abstract

**Background:**

In Japan, a variety of traditional dietary habits and daily routines have developed in many regions. The effects of these behaviors, and the regional differences in the composition of the gut microbiota, are yet to be sufficiently studied. To characterize the Japanese gut microbiota and identify the factors shaping its composition, we conducted 16S metagenomics analysis of fecal samples collected from healthy Japanese adults residing in various regions of Japan. Each participant also completed a 94-question lifestyle questionnaire.

**Results:**

We collected fecal samples from 516 healthy Japanese adults (325 females, 191 males; age, 21–88). Heatmap and biplot analyses based on the bacterial family composition of the fecal microbiota showed that subjects’ region of residence or gender were not strongly correlated with the general composition of the fecal microbiota. Although clustering analysis for the whole cohort did not reveal any distinct clusters, two enterotype-like clusters were observed in the male, but not the female, subjects.

In the whole subject population, the scores for bowel movement frequency were significantly correlated with the abundances of *Christensenellaceae*, *Mogibacteriaceae*, and *Rikenellaceae* in the fecal microbiota (*P* < 0.001). These three bacterial families were also significantly more abundant (*P* < 0.05 or 0.01) in lean subjects (body mass index (BMI) < 25) than in obese subjects (BMI > 30), which is consistent with previously published results. However, a previously reported correlation between BMI and bowel movement frequency was not observed. In addition, the abundances of these three families were positively correlated with each other and comprised a correlative network with 14 other bacterial families.

**Conclusions:**

The present study showed that the composition of the fecal microbiota of healthy Japanese adults at the national level was not strongly correlated with subjects’ area of residence or gender. In addition, enterotype partitioning was ambiguous in this cohort of healthy Japanese adults. Finally, the results implied that the abundances of *Christensenellaceae*, *Mogibacteriaceae*, and *Rikenellaceae*, along with several other bacterial components that together comprised a correlative network, contributed to a phenotype characterized by a high frequency of bowel movements and a lean body type.

**Electronic supplementary material:**

The online version of this article (doi:10.1186/s12866-016-0898-x) contains supplementary material, which is available to authorized users.

## Background

The human gut microbiota is a highly complex ecosystem composed of a large number of bacteria (10^10^–10^11^ cells/g feces) from several hundreds of bacterial species [1]. Recently, the composition of the human gut microbiota has been linked to host health and to the development of diseases such as obesity and inflammatory bowel disease [2, 3]. Therefore, modulation of the gut microbiota may be a useful means of developing personalized healthcare strategies [4].

Although variations exist in the composition of the gut microbiota from one person to another, common patterns in the bacterial ecosystem of the gut microbiota have been identified, which are called enterotypes. Arumugam et al. [5] examined the fecal microbiota of subjects from several countries and found three robust enterotypes enriched in *Bacteroides*, *Prevotella*, or *Ruminococcus* that were independent of nationality or region of residence. Subsequent studies have confirmed the existence of at least two enterotypes in which either *Prevotella* or *Bacteroides* species are predominant [6]. Host parameters such as age, gender, and body mass index (BMI) are reported to be linked to the composition of the gut microbiota [7–11], and differences in dietary habits have been shown to affect the bacterial diversity of the human gut microbiota. Together, these results partially explain why differences in the composition of the gut microbiota are observed in persons residing in different geographical regions [6, 12–14].

In Japan, a variety of regionally distinct traditional dietary habits and daily routines have developed [15]. Although the effects of dietary habits and daily routines on the composition of the fecal microbiota have been studied in small cohorts of Japanese individuals [13], these effects have not been studied at the national level. Here, we determined the abundances and prevalences of bacterial families in the fecal microbiota of a group of healthy Japanese adults residing in various regions of Japan by using a 16S metagenomic approach. We then examined whether there were any correlations between the subjects’ dietary habits and daily routines and the bacterial composition of their gut microbiota by using the results of a lifestyle questionnaire completed by the subjects.

## Methods

### Subjects, fecal sampling, and DNA extraction

Healthy adult Japanese volunteers residing in the seven regions of Japan (Hokkaido, Tohoku, Kanto, Chubu, Kansai, Chugoku-Shikoku, and Kyushu) were recruited into the study. Fecal sampling and DNA extraction were conducted by using the methods of Jin et al. [16].

### Lifestyle questionnaire

Each subject was asked to complete a questionnaire containing 94 questions about physical characteristics, dietary habits, daily routines, level of stress, and physical and mental health. To assess the subjects’ level of stress, we used 20 questions from the Center for Epidemiologic Studies Depression Scale test [17]. Additional file 1: Table S1 shows an English translation of the original Japanese questionnaire, together with the scoring system used for each question.

### Illumina sequencing and microbiological analyses

The detailed procedures for the Illumina sequencing and microbiological analyses are described in the Additional file 2. Briefly, the V1–V2 hypervariable region of the bacterial 16S rRNA gene sequence was determined by using an Illumina Miseq gene and small genome sequencer (Illumina, CA, USA). By using the Quantitative Insights Into Microbial Ecology (QIIME) bioinformatics pipeline [18], 5,290,023 high-quality reads (10,252 ± 2406 reads/sample) were generated (Additional file 1: Table S2) and assigned to operational taxonomic units (OTUs). Representative sequences for each OTU were then aligned by using the MUSCLE multiple sequence alignment algorithm [19], taxonomically classified by using RDP Classifier v2.2 [20], and then used to construct an OTU table.

### Analyses of microbial diversity

Based on the abundances of the bacterial families identified in the fecal microbiota, a heatmap and biplots were constructed, and a cluster analysis was performed by using R program version 3.1.3 [21]. The heatmap was constructed by summing the OTU table for each bacterial family. A dendrogram based on the heatmap was then drawn by using the Unweighted Pair Group Method with Arithmetic Mean (UPGMA) algorithm based on Jensen-Shannon divergence. Biplots were constructed by plotting PC1–PC2 scores obtained through a principal component analysis (PCA). Clusters were identified by conducting a principal coordinate analysis (PCoA) with the partitioning around medoids (PAM) algorithm by following the methods of Arumugam et al. [5]. Clusters with the highest Calinski-Harabasz (CH) index [22] were validated by using the methods of Koren et al. [23] to calculate prediction strength (PS) [24] and silhouette index (SI) [25]. The following thresholds were used for PS: no strong clustering (PS < 0.9), strong clustering (PS ≥ 0.9). The following thresholds were used for SI: no substantial structure (SI ≤ 0.25), weak structure (0.25 < SI ≤ 0.5), reasonable structure (0.5 < SI ≤ 0.75), and strong structure (SI > 0.75).

### Statistical analysis

All statistical analyses were conducted by using R program version 3.1.3. [21]. To compare average microbial abundances and questionnaire scores between two groups, Welch’s *t* test and the Mann-Whitney *U* test were used for numerical and ordinal data, respectively. To compare the prevalence between two groups, Fisher’s exact test was performed. To compare the prevalence among multiple groups, Holm’s correction was applied to the tests described above. Correlations among and between bacterial families and questionnaire scores were evaluated by using Kendall’s Tau-b test.

### Data deposition

The 16S metagenomic sequences determined in this study (accession number DRA005068) and the representative sequences assigned to the OTUs (accession numbers LC180363-LC183482) have been deposited in the DNA Data Bank of Japan.

## Results

### Subjects

A total of 516 healthy adult volunteers (325 females, 191 males; age, 21–88 years) was recruited into the study. The number of volunteers recruited from each region in Japan was as follows: Hokkaido, *n* = 38; Tohoku, *n* = 40; Kanto, *n* = 193; Chubu, *n* = 49; Kansai, *n* = 49; Chugoku-Shikoku, *n* = 48, and Kyushu, *n* = 99. The raw physical characteristics data for the cohort are shown in Additional file 1: Table S2. The physical characteristics of the cohort stratified by region, age, and gender are shown in Table 1. The gender ratios for each subjects’ area of residence were also shown in Additional file 3: Figure S1. Although there were no significant differences in age among the regions, there were differences in the gender ratio between some of the regions.

**Table 1:** Subject characteristics stratified by area of residence

Subject characteristics	Whole	Hokkaido	Tohoku	Kanto	Chubu	Kansai	Chugoku Shikoku	Kyushu
Subject No.	516	38	40	193	49	49	48	99
Age	52.4 ± 13.4	50.0 ± 13.8	49.7 ± 17.1	53.5 ± 12.3	53.7 ± 14.2	54.8 ± 16.1	53.3 ± 10.0	50.0 ± 13.1
Gender ratio (Female : Male)	325 : 191	14 : 24^a^	26 : 14^ab^	122 : 71^ab^	31 : 18^ab^	19 : 30^a^	36 : 12^b^	77 : 22^b^

Significance was calculated among area of residences

In each row, scores with the same letter in their superscripts or without superscripts were not significantly different (*P* ≥ 0.05) from each other

### Taxonomic analysis of Illumina sequencing data

A total of 5,290,023 high-quality reads was generated by the Illumina sequencing (Additional file 1: Table S2). These high-quality reads were assigned to 3119 OTUs that were taxonomically classified into 12 phyla, 24 classes, 39 orders, 66 families, 141 genera, 347 species, and a group of unclassified OTUs (Additional file 1: Tables S3 and S4). Good’s coverage estimate, which is an index of sampling completeness, was greater than 95% for all subjects (average, 97.9% ± 0.7%), indicating that almost all of the microbial content of the fecal samples was covered (Additional file 1: Table S2).

### Microbial diversity

**Fig. 1:**
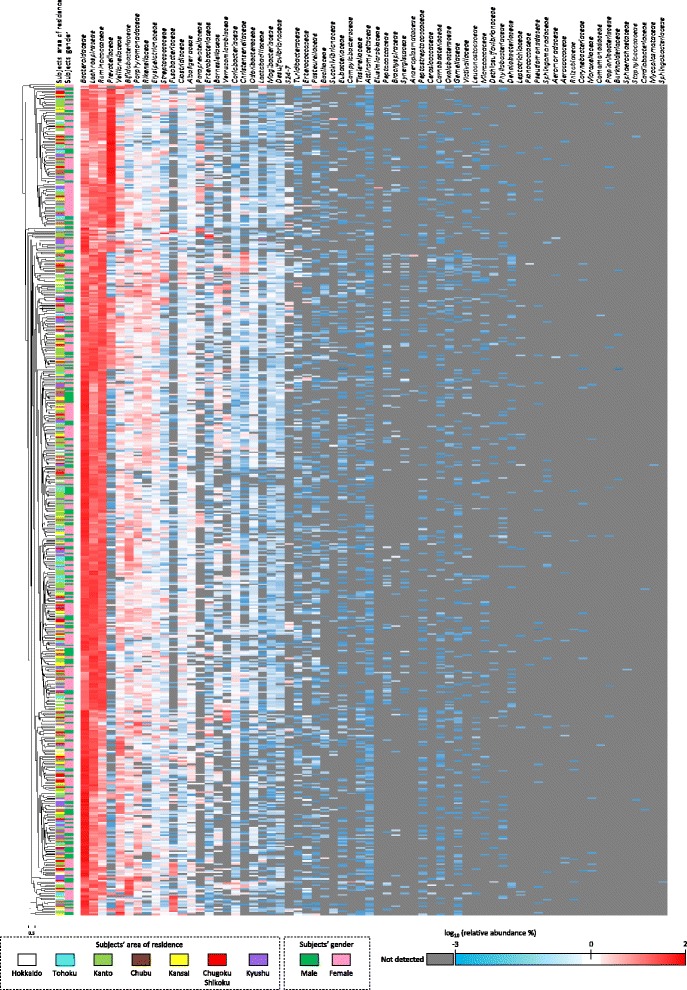
Heatmap of the abundances of the 66 bacterial families identified in the fecal microbiota. The data for each subject were aligned to a dendrogram constructed by using the UPGMA algorithm based on the Jensen-Shannon divergence. The color scale at the bottom of the heatmap shows the abundance of each bacterial family (log_10_ %) and the horizontal scale bar showed Jensen-Shannon divergence (0.5). Subjects’ area of residence and gender are indicated by using color codes on the bottom of this figure

The most predominant bacterial families in the fecal samples were *Bacteroidaceae* (mean abundance ± SD: 33.1% ± 19.0%; prevalence: 100%), *Lachnospiraceae* (17.6% ± 10.1%; 100%), *Ruminococcaceae* (15.8% ± 9.3%; 100%), and *Prevotellaceae* (9.1% ± 18.0%; 73.3%) (Additional file 1: Table S5). The heatmap constructed did not reveal any distinct clusters based on area of residence or gender (Fig. 1).

**Fig. 2:**
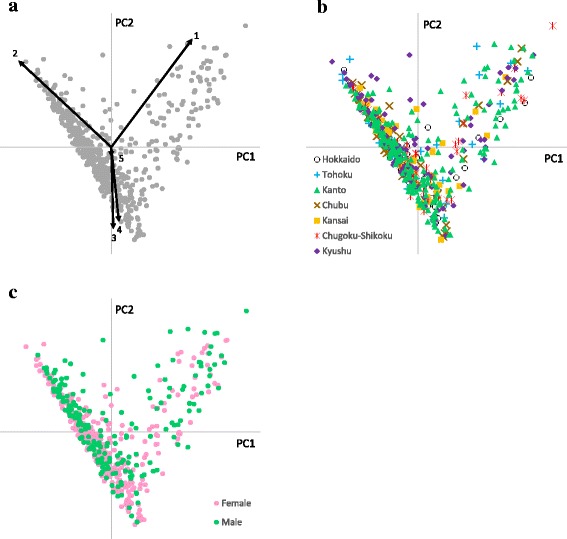
Biplots constructed based on the bacterial family composition of each subjects’ fecal microbiota. A principal component analysis of the bacterial family composition of each subjects’ fecal microbiota was performed, and a biplot in the PC1–PC2 dimension were constructed. The relative contributions of PC1 and PC2 were 54.1 and 25.7%, respectively. **a** The constructed biplot with an overlay showing the effects of the five most abundant bacterial families on plot location in the PC1–PC2 dimension (1. *Prevotellaceae*, 2. *Bacteroidaceae*, 3. *Lachnospiraceae*, 4. *Ruminococcaceae*, 5. *Bifidobacteriaceae*). **b** The biplot shown in (**a**) with the sample plots colored based on area of residence. **c** The biplot shown in (**a**) with the sample plots colored based on gender

In the biplots constructed, the location of the plots in the PC1–PC2 dimension was affected mainly by the abundances of the bacterial families *Bacteroidaceae*, *Lachnospiraceae*, *Ruminococcaceae*, and *Prevotellaceae* (hereafter referred to as “the major PCA determinants”) (Fig. 2a). Samples enriched with *Prevotellaceae* were located in the PC1-positive/PC2-positive direction. Samples enriched with *Bacteroidaceae* were located in the PC1-negative/PC2-positive direction. Samples enriched with *Lachnospiraceae* and *Ruminococcaceae* were located in the PC2-negative direction. The effect of the fifth most abundant bacterial family, *Bifidobacteriaceae*, on plot location was much smaller than those of the major PCA determinants. No distinct clusters were observed when the biplot was colored for area of residence or gender (Fig. 2b and c); however, *Lachnospiraceae* was found to be significantly more abundant (*P* < 0.05), but not more prevalent, in the subjects residing in Hokkaido compared with those residing in Kyushu (Tables 2 and 3). Significant differences (*P* < 0.05) between areas of residence were also observed in the abundance or prevalence of six other bacterial families (i.e., *Bacillaceae*, *Bifidobacteriaceae*, *Lachnospiraceae*, *Lactobacillaceae*, *Pasteurellaceae*, and S24-7), but these differences were only observed between two of the seven areas of residence for each family. No significant differences in alpha diversity score were observed among the areas of residence (Additional file 1: Table S5).

**Table 2:** Abundance of the major PCA determinants stratified by area of residence

Bacterial family	Abundance (Mean% ± SD)							
	Whole (*n* = 516)	Hokkaido (*n* = 38)	Tohoku (*n* = 40)	Kanto (*n* = 193)	Chubu (*n* = 49)	Kansai (*n* = 49)	Chugoku Shikoku (*n* = 48)	Kyushu (*n* = 99)
*Bacteroidaceae*	33.1 ± 19.0	32.4 ± 19.8	32.4 ± 19.7	30.8 ± 18.0	39.2 ± 20.1	34.7 ± 17.4	30.8 ± 17.7	35.4 ± 20.5
*Lachnospiraceae*	17.6 ± 10.1	20.3 ± 7.7^a^	18.4 ± 9.7^ab^	18.7 ± 10.5^ab^	15.3 ± 10.4^ab^	17.8 ± 9.7^ab^	18.1 ± 10.0^ab^	15.1 ± 10.0^b^
*Prevotellaceae*	9.1 ± 18.0	7.1 ± 16.8	10.0 ± 19.1	10.8 ± 19.3	5.9 ± 16.6	4.9 ± 11.1	10.4 ± 20.4	8.9 ± 17.1
*Ruminococcaceae*	15.8 ± 9.3	15.0 ± 10.3	16.5 ± 9.9	16.4 ± 8.9	15.3 ± 10.1	15.1 ± 9.9	15.0 ± 9.0	15.9 ± 8.8

Significance was calculated among area of residences

In each row, scores with the same letter in their superscripts or without superscripts were not significantly different (*P* ≥ 0.05) from each other

**Table 3:** Prevalence of the major PCA determinants stratified by area of residence

Bacterial family	Prevalence (%)							
	Whole (*n* = 516)	Hokkaido (*n* = 38)	Tohoku (*n* = 40)	Kanto (*n* = 193)	Chubu (*n* = 49)	Kansai (*n* = 49)	Chugoku Shikoku (*n* = 48)	Kyushu (*n* = 99)
*Bacteroidaceae*	100.0	100.0	100.0	100.0	100.0	100.0	100.0	100.0
*Lachnospiraceae*	100.0	100.0	100.0	100.0	100.0	100.0	100.0	100.0
*Prevotellaceae*	73.3	76.3	67.5	74.1	69.4	65.3	77.1	76.8
*Ruminococcaceae*	100.0	100.0	100.0	100.0	100.0	100.0	100.0	100.0

Significance was calculated among area of residences

In each row, scores without superscripts were not significantly different (*P* ≥ 0.05) from each other

Among the major PCA determinants, the abundance of *Prevotellaceae* was significantly higher (*P* < 0.01) in male subjects, and the abundance of *Ruminococcaceae* was significantly higher (*P* < 0.01) in female subjects (Table 4). Overall, significant differences (*P* < 0.05) in the abundance or prevalence of 24 of the 66 identified bacterial families were observed between female and male subjects (Additional file 1: Table S6). Compared with the male subjects, the female subjects also had significantly higher alpha diversity scores (Chao1 index, *P* < 0.05; Shannon index, *P* < 0.01; phylogenetic diversity whole tree, *P* < 0.01) (Additional file 1: Table S6).

**Table 4:** Abundance and prevalence of the major PCA determinants stratified by gender

Bacterial family	Abundance (Mean% ± SD)			Prevalence (%)		
	Female (*n* = 325)	Male (*n* = 191)	Significance	Female (*n* = 325)	Male (*n* = 191)	Significance
*Bacteroidaceae*	33.2 ± 18.5	32.9 ± 19.7	NS	100	100	NS
*Lachnospiraceae*	18.0 ± 10.4	17.0 ± 9.5	NS	100	100	NS
*Prevotellaceae*	9.7 ± 17.7	16.6 ± 22.6	**	71.1	77.0	NS
*Ruminococcaceae*	17.9 ± 8.7	12.4 ± 9.2	**	100	100	NS

NS: *P* ≥ 0.05, **: *P* < 0.01

**Fig. 3:**
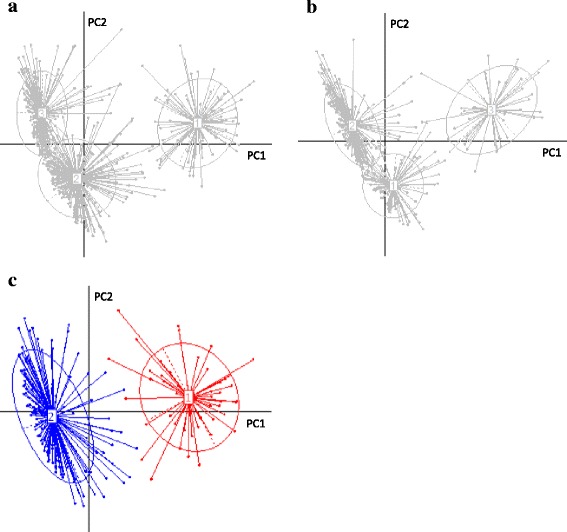
Cluster analysis based on the bacterial family compositions of the microbiota. Clusters were identified by conducting a PCoA with the PAM algorithm for the whole cohort (**a**) and for the female (**b**) and the male (**c**) subjects based on the bacterial family composition of the microbiota. The relative contributions of PC1 versus PC2 for (**a**), (**b**), and (**c**) were 35.4% versus 18.4%, 32.6% versus 18.8%, and 40.1% versus 17.2%, respectively. In (**c**), the different colors indicate that the clusters were statistically reliable

In the cluster analysis, no statistically reliable clusters (i.e., clusters with PS > 0.9 and SI > 0.25) were found when the whole subjects’ data were used. To examine whether there were any trends in the cluster partitioning, cluster analyses were also conducted for each subjects’ area of residence and gender. When only the data for the male subjects were analyzed, two statistically reliable, weakly partitioned clusters (PS = 0.96, SI = 0.34) were observed (Table 5 and Fig. 3). Between these two clusters, significant differences in abundance or prevalence were observed for 21 of the 66 identified bacterial families (Additional file 1: Table S7). Among the major PCA determinants, the abundance and prevalence of *Prevotellaceae* was significantly higher (*P* < 0.01) in cluster 1 compared with the other families in the cluster, whereas the abundances of *Bacteroidaceae* (*P* < 0.01), *Lachnospiraceae* (*P* < 0.01), and *Ruminococcaceae* (*P* < 0.05) were significantly higher in cluster 2 compared with the other families in the cluster (Additional file 1: Table S7).

**Table 5:** Statistical output for the cluster analysis

Statistics	Whole	Female	Male	Hokkaido	Tohoku	Kanto	Chubu	Kansai	Chugoku Shikoku	Kyushu
Cluster no. showing max CH score	3	3	2	2	2	3	4	2	2	3
Max CH score	328	183	208	21	30	186	32	33	44	60
Prediction strength	0.62	0.59	0.96	0.62	0.67	0.56	0.51	0.61	0.68	0.63
Silhouette index	0.20	0.20	0.34	0.32	0.36	0.21	0.20	0.16	0.36	0.20

### Questionnaire scores

The raw questionnaire data are presented in Additional file 1: Table S8. There were significant differences (*P* < 0.05) in scores for some of the questions among the areas of residence and between the genders (Additional file 1: Tables S9 and S10).

### Correlations between microbial families abundance and questionnaire score

Significant differences (*P* < 0.01 or *P* < 0.05) were observed between the two clusters in the male subjects for the scores for one question about dietary habits and for four questions about physical and mental health (Table 6 and Additional file 1: Table S11). The scores for “Mushroom intake frequency” (Q 29) and “I feel like I am becoming quite healthy” (Q 94) were significantly higher in cluster 1 than in cluster 2, whereas the scores for “I was bothered by things that usually do not bother me within the past week” (Q 52), “A close relationship was negatively affected by a physical or mental health problem within the past month” (Q 81), and “I felt nervous within the past month” (Q 83) were significantly higher in cluster 2 than in cluster 1. However, there were no significant correlations between the scores to these questions and the abundance of any bacterial family.

**Table 6:** Question scores that were significantly different between the two clusters identified in the male subjects

Question		Cluster 1 (Mean ± SD)	Cluster 2 (Mean ± SD)	Significance
Q 29	Mushroom intake frequency	2.6 ± 0.8	2.1 ± 0.6	**
Q 52	I was bothered by things that usually do not bother me within the past week	1.2 ± 0.5	1.5 ± 0.7	*
Q 81	A close relationship was negatively affected by a physical or mental health problem within the past month	1.3 ± 0.6	1.5 ± 0.9	*
Q 83	I felt nervous within the past month	1.8 ± 0.9	2.1 ± 1.0	*
Q 94	I feel like I am becoming quite healthy	3.8 ± 0.9	3.5 ± 0.9	*

NS: *P* ≥ 0.05, *: *P* < 0.05, **: *P* < 0.01

**Fig. 4:**
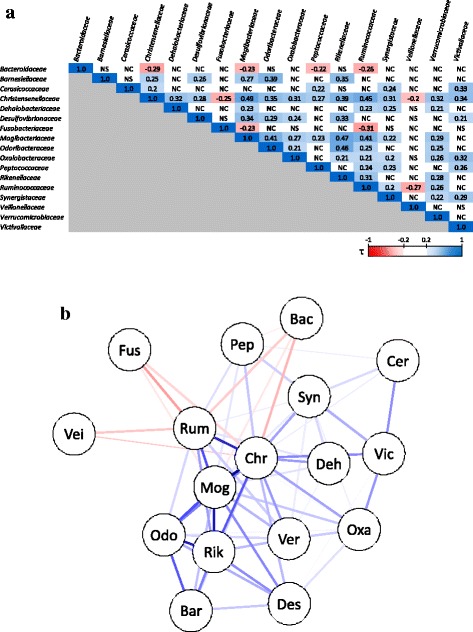
Correlations between *Christensenellaceae*, *Mogibacteriaceae*, *Rikenellaceae*, and 14 other bacterial families. Correlations between the abundance of *Christensenellaceae*, *Mogibacteriaceae*, *Rikenellaceae*, and 14 other bacterial families are shown as a heatmap (**a**) and network (**b**). **a** Kendall’s Tau-b ratio is shown for combinations with *P* < 0.001 and |τ| > 0.2. The strength of the shading within each cell indicates the strength of the positive (*blue*) or negative (*red*) correlation for a given bacterial family combination. NS, *P* ≥ 0.001; NC, |τ| ≤ 0.2. **b** Nodes for two correlated families (*P* < 0.001 and |τ| > 0.2) are shown connected by a *line*. The color, strength, and thickness of the lines indicate the strength of the positive (*blue*) or negative (*red*) correlation between the families. Bac, *Bacteroidaceae*; Bar, *Barnesiellaceae*; Cer, *Cerasicoccaceae*; Chr, *Christensenellaceae*; Deh, *Dehalobacteriaceae*; Des, *Desulfovibrionaceae*; Fus, *Fusobacteriaceae*; Mog, *Mogibacteriaceae*; Odo, *Odoribacteraceae*; Oxa, *Oxalobacteraceae*; Pep, *Peptococcaceae*; Rik, *Rikenellaceae*; Rum, *Ruminococcaceae*; Syn, *Synergistaceae*; Vei, *Veillonellaceae*; Ver, *Verrucomicrobiaceae*; Vic, *Victivallaceae*

There were significant weak negative correlations between bowel movement frequency (Q 7) and the abundance of *Christensenellaceae*, *Mogibacteriaceae*, and *Rikenellaceae* (*P* < 0.001, τ = −0.24, −0.28, and −0.25, respectively) (Table 7). There were significant positive correlations (*P* < 0.001 and τ = 0.39–0.49) among the abundances of these families (Fig. 4a). The abundance of at least one of these families was significantly correlated (*P* < 0.001 and |τ| > 0.2) with that of at least one of 14 other bacterial families (Fig. 4b), among which 11 families had a positive correlation (*Barnesiellaceae*, *Cerasicoccaceae*, *Dehalobacteriaceae*, *Desulfovibrionaceae*, *Odoribacteraceae*, *Oxalobacteraceae*, *Peptococcaceae*, *Ruminococcaceae*, *Synergistaceae*, *Verrucomicrobiaceae*, and *Victivallaceae*), and three families had a negative correlation (*Bacteroidaceae*, *Fusobacteriaceae*, and *Veillonellaceae*). In addition, there were significant weak positive correlations between the score for frequency of dairy product intake frequency (Q 13) and abundance of *Lactobacillaceae* (*P* < 0.001, τ = 0.25), and between the score for frequency of *natto* (traditional Japanese fermented soy beans) intake frequency (Q 21) and the abundance of *Bacillaceae* (*P* < 0.001, τ = 0.34) (Table 7).

**Table 7:** Question scores that were significantly correlated with bacterial family abundance

Question		Bacterial family	*P*	τ	Abundance (Mean% ± SD) (*n* = 516)	Prevalence (%) (*n* = 516)
Q 7	Bowel movement frequency	*Christensenellaceae*	<0.001	−0.24	0.39 ± 1.54	56.8
Q 7	Bowel movement frequency	*Mogibacteriaceae*	<0.001	−0.28	0.18 ± 0.27	82.4
Q 7	Bowel movement frequency	*Rikenellaceae*	<0.001	−0.25	1.51 ± 1.82	90.9
Q 13	Dairy product intake frequency	*Lactobacillaceae*	<0.001	0.25	0.19 ± 1.35	52.5
Q 21	*Natto* intake frequency	*Bacillaceae*	<0.001	0.34	0.03 ± 0.36	26.6

Combinations showing significant correlation (*P* < 0.001 and |τ| > 0.2) were listed

The abundance of two OTUs belonging to *Christensenellaceae* (OTUs 844 and 2858) and of one OTU belonging to *Mogibacteriaceae* (OTU 417) had a weak negative correlation with the score for bowel movement frequency (Q 7, τ = −0.21, −0.22, and −0.23, respectively) (Table 8). The representative sequences for these OTUs had low identities with the sequences of known species (80.7–81.9%, 80.6–81.4%, and 82.0–87.5%, respectively) and therefore could not be identified at the species level (Additional file 1: Table S12). However, the abundance of one species belonging to family *Lactobacillaceae* (OTU 318) had a weak positive correlation with the score for frequency of dairy product intake frequency (Q 13, τ = 0.36), and the abundance of one species belonging to family *Bacillaceae* (OTU 547) had a weak positive correlation with the score for intake frequency of *natto* (Q 21, τ = 0.36). The representative sequences for these OTUs had a high identity with known sequences of species belonging to the *Lactobacillus casei* [26] and *Bacillus subtilis* subgroups [27], respectively.

**Table 8:** Correlations between question score and operational taxonomic unit abundance

Question		OTU ID	Bacterial family	*P*	τ	Abundance (Mean% ± SD) (*n* = 516)	Prevalence (%) (*n* = 516)
Q 7	Bowel movement frequency	844	*Christensenellaceae*	<0.001	−0.21	0.05 ± 0.03	14.3
Q 7	Bowel movement frequency	2858	*Christensenellaceae*	<0.001	−0.22	0.03 ± 0.01	11.6
Q 7	Bowel movement frequency	417	*Mogibacteriaceae*	<0.001	−0.23	0.04 ± 0.03	18.0
Q 13	Dairy product intake frequency	318	*Lactobacillaceae*	<0.001	0.36	0.09 ± 0.08	24.8
Q 21	*Natto* intake frequency	547	*Bacillaceae*	<0.001	0.36	0.05 ± 0.03	25.2

Combinations showing significant correlation (*P* < 0.001 and |τ| > 0.2) were listed

### Correlations between BMI and bowel movement frequency or abundance of the three bacterial families correlated with bowel movement frequency

Although bowel movement frequency in lean (BMI < 25, *n* = 425) and in obese (BMI > 30, *n* = 13) subjects was comparable, the abundances of the three bacterial families correlated with bowel movement frequency were significantly higher in lean subjects than in obese subjects (*Christensenellaceae* and *Mogibacteriaceae*, *P* < 0.01; *Rikenellaceae*, *P* < 0.05). The prevalence of *Christensenellaceae* was also significantly higher in lean subjects than in obese patients (*P* < 0.05) (Table 9).

**Table 9:** Bowel movement frequency and correlated bacterial families in lean and obese subjects

	Questionnaire score (Mean ± SD) or abundance (Mean% ± SD)			Prevalence (%)		
	Lean group (BMI < 25) (*n* = 425)	Obese group (BMI > 30) (*n* = 13)	Significance	Lean group (BMI < 25) (*n* = 425)	Obese group (BMI > 30) (*n* = 13)	Significance
Bowel movement frequency	3.53 ± 0.77	3.54 ± 0.78	NS	-	-	-
*Christensenellaceae*	0.77 ± 2.14	0.01 ± 0.004	**	59.3	23.1	*
*Mogibacteriaceae*	0.23 ± 0.29	0.06 ± 0.05	**	84.7	69.2	NS
*Rikenellaceae*	1.77 ± 1.86	0.90 ± 0.96	*	91.5	84.6	NS

NS: *P* ≥ 0.05, *: *P* < 0.05, **: *P* < 0.01

## Discussion

### Microbial diversity in the fecal microbiota

In the biplot analysis based on the bacterial family composition of the fecal microbiota, the location of the plots in the PC1–PC2 dimension was affected mainly by the abundances of *Prevotellaceae*, *Bacteroidaceae*, *Lachnospiraceae*, and *Ruminococcaceae*, which are the same families that were found to be driving the enterotypes identified in a previous study [5] (Fig. 2a). Furthermore, no distinct clusters were found in the data from the whole subjects (Table 5 and Fig. 3a), which is consistent with previous studies [23, 28]. This suggests that the more sample data is used, the less distinct the enterotype partitioning becomes.

### Association between area of residence and the bacterial composition of the fecal microbiota

No distinct clusters were found for area of residence in the heatmap of bacterial family composition (Fig. 1) or in the biplots of the PC1–PC2 dimension (Fig. 2b). Furthermore, differences were found for the abundances of only six bacterial families between only two of the seven regions (Table 2 and Additional file 1: Table S5), suggesting that area of residence does not strongly affect the composition of the fecal microbiota in the healthy adults living in Japan. Therefore, the dietary habits and daily routines that are common in the various regions of Japan today (Additional file 1: Table S9) are not sufficiently distinct as to lead to clear microbial diversity in the gut, which is in contrast to the current situation in Thailand [14] and Mongolia [10].

### Cluster analysis based on bacterial family composition and gender

Although no significant differences in the composition of the fecal microbiota were found between the female and male subjects in the heatmap and biplots (Figs 1 and 2c), statistically reliable clusters were observed in the male subjects but not in the female subjects (Table 5 and Fig. 3b, c). Furthermore, significant differences were found between female and male subjects in the abundance or prevalence of the 66 identified microbial families (Additional file 1: Table S6). Given that the distributions of the plots constructed by the PCA and PCoA were comparable, the position of the plots in the PCoA should reflect the abundances of the major PCA determinants (Figs 2a and 3). In male subjects, the abundance and prevalence of *Prevotellaceae*, which was the sole strong determinant of a sample plot being located in the PC1-positive/PC2-positive direction in the PCA analysis, were both significantly higher (*P* < 0.01) than in female subjects (Table 4), which may account for the distinct partitioning between cluster 1 and cluster 2 in the male subjects. Recent studies have demonstrated that the composition of the gut microbiota is gender-specific (microgenderome) and that there is a gender-specific, bidirectional relationship between the host and the gut microbiota that is mediated via sex hormones [7, 29]. This suggests that the differences in the composition of the fecal microbiota observed between the female and male subjects in the present study are a result of a gender-specific relationship between the host and their gut microbiota. Since there were significant differences in some of the question scores between the female and male subjects (Additional file 1: Table S10), it is also possible that differences in lifestyle between the female and male subjects contributed to the differences observed in the bacterial composition of the fecal microbiota.

### Meaning of the clusters observed in the male subjects

Although the scores for one question about food intake frequency and for four questions about physical and mental health were significantly different between the two clusters in the male subjects (Table 6), these scores were not correlated with the abundance of any bacterial family. This suggests that food intake frequency does not strongly affect the composition of the fecal microbiota and that the composition of the fecal microbiota does not strongly affect the host’s physical and mental health. Indeed, the differences observed in the scores for the questions related to physical and mental health may simply be a result of environmental or genetic factors.

### Effects of dietary habits on the bacterial composition of the fecal microbiota

Among the questions examining dietary habits, the frequencies of dairy product and *natto* intake were significantly correlated (*P* < 0.001) with the abundances of one *Lactobacillaceae* species and of one *Bacillaceae* species (Table 7). Given that some species of *Lactobacillus*, which is the main genus in family *Lactobacillaceae*, are used in the manufacture of dairy products [30], and that *natto* is a product made by fermenting soybeans with *B. subtilis* [31], it is not surprising that the frequencies of intake of these products were positively correlated with the abundances of these bacterial families. Indeed, the OTUs assigned to these two species were classified into the *L. casei* subgroup to which some probiotic strains belong [32] and the *B. subtilis* subgroup. In the present study, the frequency of intake of only two kinds of foodstuffs were correlated with the abundance of a bacterial family, implying that the human gut microbiota is affected more by the amount of particular foods consumed rather than by the frequency of intake.

### Relationship between BMI and bacterial family abundance or bowel movement frequency

The abundances of *Christensenellaceae*, *Mogibacteriaceae*, and *Rikenellaceae* were negatively correlated with bowel movement frequency (Table 7). Furthermore, the abundances of these bacterial families were significantly higher in lean subjects (BMI < 25) than in obese subjects (BMI > 30) (Table 9), which is consistent with previous results [9]. Furthermore, the abundances of *Christensenellaceae*, *Mogibacteriaceae*, and *Rikenellaceae* were correlated with each other and formed part of a positively correlated network together with 11 other bacterial families. Three negatively correlated families (*Bacteroidaceae*, *Fusobacteriaceae*, and *Veillonellaceae*) were significantly correlated with four of the components of this network (*Christensenellaceae*, *Mogibacteriaceae, Peptococcaceae*, and *Ruminococcaceae*) (Fig. 4), indicating that BMI and bowel movement frequency could be controlled by modulating the abundances of *Christensenellaceae*, *Mogibacteriaceae*, and *Rikenellaceae*. As previous study reported, the abundances of these families associated with lower level of triglyceride and higher level of HDL cholesterol both of which correlate to lower BMI [33]. The abundances of *Christensenellaceae* and *Rikenellaceae* were highly associated with lower triglyceride, while the abundance *Mogibacteriaceae* of was highly associated with the higher level of HDL, implying their underlining mechanisms to reduce BMI were different. In addition, a cultured member of *Christensenellaceae* reduced weight and adiposity gains with amending obese associated microflora when inoculated to germ-free mice [9]. This result suggested that *Christensenellaceae* was one of the functional key factors contributing to lean body type in the correlation network. However, since there is little published work and this study was based on the 16S rRNA gene profiles, therefore, the further investigation should be needed to explain the functional role of these bacterial families to modify the host’s bowel movement frequency. Although these three families did not have a direct correlation in a study by Goodrich et al. [9], they were positively correlated with each other, as in our study, in the Yatsunenko dataset described in the same report, which suggests that these correlations are cohort-dependent.

A positive correlation between BMI and bowel movement frequency has been previously reported in a large cohort of European subjects, and it was also reported that obese subjects had a significantly higher bowel movement frequency compared with lean subjects [34]. However, in the present study, no significant differences in bowel movement frequency were found between lean and obese subjects (*P* = 0.74, τ = 0.01) (Table 9), suggesting that the correlation between bowel movement frequency and the abundance of *Christensenellaceae*, *Mogibacteriaceae*, and *Rikenellaceae* observed in the present study was not solely a result of BMI. The abundance of OTUs belonging to the families *Christensenellaceae* (OTUs 844 and 2858) and *Mogibacteriaceae* (OTU 417) were also correlated with bowel movement frequency. However, given that the abundances of these OTUs were relatively low compared with those of the other OTUs in the same bacterial family, the correlation between the abundance of these bacterial families and bowel movement frequency was likely not a result of the abundance of these specific OTUs but of the combined abundance of all of the OTUs in the families. A similar relationship with bowel movement frequency was found for *Rikenellaceae*: however, no OTUs belonging to this family were correlated with bowel movement frequency.

## Conclusions

In the present study, we used a 16S metagenomics method to characterize the fecal microbiota of 516 healthy Japanese adults living in various regions of Japan. Based on the bacterial family composition of the fecal microbiota, subjects’ region of residence and gender were not strongly correlated with the general composition of the fecal microbiota. Clustering analysis showed that the enterotype partitioning was ambiguous in the whole cohort. However, two enterotype-like clusters were observed in the male, but not the female, subjects, suggesting that the composition of the fecal microbiota has a gender-specific component. The bacterial compositions of the microbiota were then compared with scores obtained from a lifestyle questionnaire completed by each subject. Significant correlations were found between bowel movement frequency and the abundances of *Christensenellaceae*, *Mogibacteriaceae*, and *Rikenellaceae*. Furthermore, the abundance of these bacterial families was significantly higher in lean subjects than in obese subjects. The abundances of these families were positively correlated with each other and comprised a correlative network with 14 other bacterial families. Together, our results present an overview of the microbial composition of the fecal microbiota of healthy Japanese adults residing in Japan and implied that the abundances of *Christensenellaceae*, *Mogibacteriaceae*, and *Rikenellaceae* contributed subject’s high bowel movement frequency and lean phenotype together with those of some other bacterial components comprising correlative network with them.

## Additional files

Additional file 1: Table S1. English translation of the lifestyle questionnaire and the scoring system used for each question. **Table S2.** Subject characteristics and the results of the Illumina sequencing. **Table S3.** Operational taxonomic unit table for the whole subjects (% in whole reads). **Table S4.** Basic Local Alignment Search Tool (BLAST) results and representative sequences for the operational taxonomic units. **Table S5.** Abundance and prevalence of the 66 identified bacterial families and alpha-diversity scores stratified by area of residence. **Table S6.** Abundance and prevalence of the 66 identified bacterial families and alpha-diversity scores stratified by gender. **Table S7.** Abundance and prevalence of the 66 identified bacterial families and alpha-diversity scores in the two clusters identified in the male subjects. **Table S8.** Raw questionnaire data. **Table S9.** Questionnaire scores stratified by area of residence. **Table S10.** Questionnaire scores stratified by gender. **Table S11.** Questionnaire scores in the two clusters identified in the male subjects. **Table S12.** Detailed Basic Local Alignment Search Tool (BLAST) search results for the operational taxonomic units shown in Table 8. **Table S13.** Quantitative Insights Into Microbial Ecology (QIIME) bioinformatics pipeline scripts used in the present study. (XLSX 8232 kb)
Additional file 2: Detailed methods for the Illumina sequencing and microbiological analyses. (DOCX 36 kb)
Additional file 3: Figure S1. Distribution of subjects’ genders stratified by area of residence. Gender ratios of female to male represent in brackets. Among the areas sharing the same letter above their bar graphs, significant difference was not observed (*P* ≥ 0.05) in the gender ratio. (PPTX 144 kb)
