# Supplementary material for: Comprehensive analysis of the fecal microbiota of healthy Japanese adults reveals a new bacterial lineage associated with a phenotype characterized by a high frequency of bowel movements and a lean body type
Source: BMC Microbiol. 2016 Nov 28;16:284. doi: 10.1186/s12866-016-0898-x (PMC5127096; doi:10.1186/s12866-016-0898-x)
Supplement: Additional file 2: — Detailed methods for the Illumina sequencing and microbiological analyses. (DOCX 36 kb) [file 12866_2016_898_MOESM2_ESM.docx]

Additional file 2: Methods

*16S rRNA amplification and sequencing*

27Fmod2-MiSeqV2 and 338RMiSeqV2 [1] were used as forward and reverse primers, respectively. For the polymerase chain reaction, Illumina adapter sequences were attached to the 3’ ends of both primers, and 12 bp of error-correcting Golay barcode sequences were inserted into the Illumina adapter sequence attached to the reverse primer to facilitate sample assignment. The bacterial 16S rRNA gene was amplified by following the methods of Kim et al. (2013) [2], and amplicons were purified with an AMPure XP Kit (Beckman Coulter, Brea, CA, USA). After quantification of the dsDNA concentration by using a Quant-iTTM PicoGreen dsDNA Assay Kit (Invitrogen, Carlsbad, CA, USA), equal amounts of dsDNA and each amplicon were pooled. The pooled dsDNA was mixed with the same amount of PhiX control (Illumina) and sequenced by using a Miseq Reagent kit v2 (500 cycles) (Illumina).

*Data processing*

All procedures were conducted with the Quantitative Insights Into Microbial Ecology (QIIME) bioinformatics pipeline v1.8.0 [3]. Detailed settings and references for the scripts used in the study are shown in Supplemental Table S12. Read data obtained for the forward and reverse primers was connected with the join_paired_ends.py script (settings: minimal overlap, 120 bp; maximal mismatch in overlap region, 8%). A quality check was conducted by using the split_libraries_fastq.py script against the 5,907,418 reads (11,448 ± 2624 reads/sample) generated in the former process, and reads that had incomplete forward primer sequences or a sequence with a quality score of less than 25 were filtered. During this process, reads were also assigned to the samples based on the barcode sequences. To filter reads with incomplete reverse primer sequences, the truncate_reverse_primer.py script was applied. The pick_otus.py script, which uses the USEARCH algorithm v5.2 [4], was used to construct operational taxonomic units (OTUs) composed of reads with more than 97% sequence similarity with each other and to remove OTUs composed of a single read (singletons). OTUs composed of reads with more than 97% sequence similarity against Phi X control were removed with the exclude_seqs_by_blast.py script. Finally, 5,290,023 high-quality reads (10,252 ± 2406 reads/sample) were grouped into 3120 OTUs, and the most abundant sequences of each OTU were selected as representative sequences by using the pick_rep_set.py script.

## *Taxonomic analysis and alpha diversity analysis*

Alignment and taxonomic classification processes were conducted with the QIIME bioinformatics pipeline v1.8.0 [3]. Representative sequences for each OTU were aligned with the align_seqs.py script and the MUSCLE algorithm [5]. A phylogenetic tree was then constructed the aligned representative sequences by using the make_phylogeny.py script and the MUSCLE algorithm. The representative sequences were taxonomically classified with the parallel_assign_taxonomy_rdp.py script and RDP classifier v2.2 [6] against the GreenGenes 13_8 97% OTU Representative Sequences Database [7]. An OTU table was constructed with the make_otu_table.py script. For detailed species identification, the representative sequences for each OTU were identified by means of a Basic Local Alignment Search Tool (BLAST) v2.2.29+ against the All-Species Living Tree databases (release LTP119) [8], with the threshold for sequence identity set at 97%. To determine the alpha diversity, 4114 representative reads were randomly selected for each sample and the alpha_rarefaction.py script was used to calculate the chao1 index [9], Shannon index [10], phylogenetic diversity [11], and Good’s coverage estimate [12].

References

1. Kato-Kataoka A, Nishida K, Takada M, Kawai M, Kikuchi-Hayakawa H, Suda K, et al. Fermented milk containing Lactobacillus casei strain Shirota preserves the diversity of the gut microbiota and relieves abdominal dysfunction in healthy medical students exposed to academic stress. Appl Environ Microbiol. 2016;AEM.04134-15.

2. Kim SW, Suda W, Kim S, Oshima K, Fukuda S, Ohno H, et al. Robustness of gut microbiota of healthy adults in response to probiotic intervention revealed by high-throughput pyrosequencing. DNA Res. 2013;20:241-53.

3. Caporaso J, Kuczynski J, Stombaugh J, Bittinger K, Bushman F, Costello E, et al. QIIME allows analysis of high-throughput community sequencing data. Nat Methods. 2010;7:335-6.

4. Edgar RC. Search and clustering orders of magnitude faster than BLAST Robert C. Bioinforma Appl Note. 2010;26:2460-1.

5. Edgar RC. MUSCLE: a multiple sequence alignment method with reduced time and space complexity. BMC Bioinformatics. 2004;5:113.

6. Cole JR, Chai B, Farris RJ, Wang Q, Kulam-Syed-Mohideen AS, McGarrell DM, et al. The ribosomal database project (RDP-II): introducing myRDP space and quality controlled public data. Nucleic Acids Res. 2007;35:D169-72.

7. DeSantis TZ, Hugenholtz P, Larsen N, Rojas M, Brodie EL, Keller K, et al. Greengenes, a chimera-checked 16S rRNA gene database and workbench compatible with ARB. Appl Environ Microbiol. 2006;72:5069-72.

8. Quast C, Pruesse E, Yilmaz P, Gerken J, Schweer T, Yarza P, et al. The SILVA ribosomal RNA gene database project: improved data processing and web-based tools. Nucleic Acids Res. 2013;41:D590-6.

9. Chao A. Nonparametric estimation of the number of classes in population. Scand J Stat. 1984;11:265-70.

10. Shannon CE. The mathematical theory of communication. Bell Syst Tech J. 1948;27:623-56.

11. Faith DP. Conservation evaluation and phylogenetic diversity. Biol Conserv. 1992;61:1-10.

12. Good I. The population frequencies of species and the estimation of population parameters. Biometrika. 1953;40:237-64.
