# Supplementary material for: Comprehensive analysis of the fecal microbiota of healthy Japanese adults reveals a new bacterial lineage associated with a phenotype characterized by a high frequency of bowel movements and a lean body type
Source: BMC Microbiol. 2016 Nov 28;16:284. doi: 10.1186/s12866-016-0898-x (PMC5127096; doi:10.1186/s12866-016-0898-x)
Supplement: Additional file 3: Figure S1. — Distribution of subjects’ genders stratified by area of residence. Gender ratios of female to male represent in brackets. Among the areas sharing the same letter above their bar graphs, significant difference was not observed (P ≥ 0.05) in the gender ratio. (PPTX 144 kb) [file 12866_2016_898_MOESM3_ESM.pptx]

## Slide 1
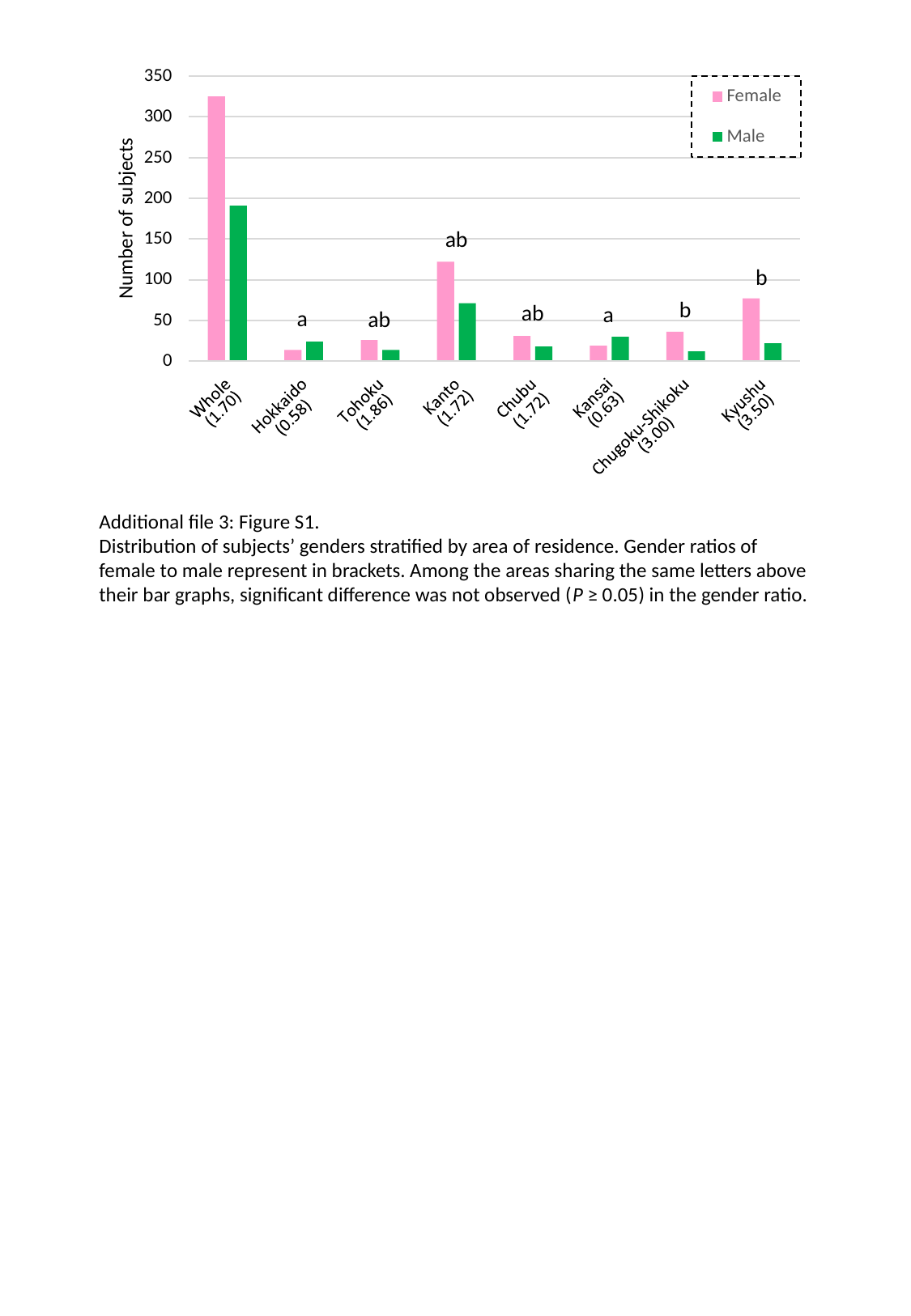

Additional file 3: Figure S1.
Distribution of subjects’ genders stratified by area of residence. Gender ratios of female to male represent in brackets. Among the areas sharing the same letters above their bar graphs, significant difference was not observed (P ≥ 0.05) in the gender ratio.
